# Longitudinal associations of long-term exposure to ultrafine particles with blood pressure and systemic inflammation in Puerto Rican adults

**DOI:** 10.1186/s12940-018-0379-9

**Published:** 2018-04-05

**Authors:** Laura Corlin, Mark Woodin, Jaime E. Hart, Matthew C. Simon, David M. Gute, Joanna Stowell, Katherine L. Tucker, John L. Durant, Doug Brugge

**Affiliations:** 1 Department of Civil and Environmental Engineering, Tufts University, 200 College Avenue, Medford, MA 02155 USA; 2 Department of Public Health and Community Medicine, Tufts University, 145 Harrison Ave, Boston, MA 02111 USA; 3 Channing Division of Network Medicine, Brigham and Women’s Hospital and Harvard Medical School, 401 Park Drive, Landmark 3rd Floor West, Boston, MA 02215 USA; 4 Department of Environmental Health, Harvard T.H. Chan School of Public Health, 401 Park Drive, Landmark 3rd Floor West, Boston, MA 02215 USA; 5 Department of Biomedical and Nutritional Sciences, University of Massachusetts-Lowell, 3 Solomont Way Suite 4, Lowell, MA 01854 USA; 6 Tisch College of Civic Life, Tufts University, 10 Upper Campus Rd, Medford, MA 02155 USA

**Keywords:** Ultrafine particulate matter, Blood pressure, C-reactive protein, Susceptible populations, Exposure assessment, Hispanic

## Abstract

**Background:**

Few longitudinal studies have examined the association between ultrafine particulate matter (UFP, particles < 0.1 μm aerodynamic diameter) exposure and cardiovascular disease (CVD) risk factors. We used data from 791 adults participating in the longitudinal Boston Puerto Rican Health Study (Massachusetts, USA) between 2004 and 2015 to assess whether UFP exposure was associated with blood pressure and high sensitivity C-reactive protein (hsCRP, a biomarker of systemic inflammation).

**Methods:**

Residential annual average UFP exposure (measured as particle number concentration, PNC) was assigned using a model accounting for spatial and temporal trends. We also adjusted PNC values for participants’ inhalation rate to obtain the particle inhalation rate (PIR) as a secondary exposure measure. Multilevel linear models with a random intercept for each participant were used to examine the association of UFP with blood pressure and hsCRP.

**Results:**

Overall, in adjusted models, an inter-quartile range increase in PNC was associated with increased hsCRP (β = 6.8; 95% CI = − 0.3, 14.0%) but not with increased systolic blood pressure (β = 0.96; 95% CI = − 0.33, 2.25 mmHg), pulse pressure (β = 0.70; 95% CI = − 0.27, 1.67 mmHg), or diastolic blood pressure (β = 0.55; 95% CI = − 0.20, 1.30 mmHg). There were generally stronger positive associations among women and never smokers. Among men, there were inverse associations of PNC with systolic blood pressure and pulse pressure. In contrast to the primary findings, an inter-quartile range increase in the PIR was positively associated with systolic blood pressure (β = 1.03; 95% CI = 0.00, 2.06 mmHg) and diastolic blood pressure (β = 1.01; 95% CI = 0.36, 1.66 mmHg), but not with pulse pressure or hsCRP.

**Conclusions:**

We observed that exposure to PNC was associated with increases in measures of CVD risk markers, especially among certain sub-populations. The exploratory PIR exposure metric should be further developed.

**Electronic supplementary material:**

The online version of this article (10.1186/s12940-018-0379-9) contains supplementary material, which is available to authorized users.

## Background

Long-term exposure to fine particulate matter (PM_2.5_, < 2.5 μm aerodynamic diameter) has been associated with cardiovascular disease (CVD) risk factors, such as increased blood pressure (BP) and concentrations of biomarkers of systemic inflammation, as well as increased risk of hypertension [1–6]. Less is known, however, about the impact of the smallest size fraction of PM, ultrafine PM (UFP, < 0.1 μm aerodynamic diameter), on these indicators. Compared to larger size fractions, UFP has a larger total deposition fraction, can penetrate more deeply into the lungs, has greater total surface area with which to interact with epithelial cells, is more likely to cross biological barriers, and can induce oxidative stress more readily [7–15].

A major source of UFP in urban areas is motor vehicle exhaust and there is high spatial and temporal variability of UFP compared to other sizes of particulate matter, especially near roadways [16]. Due in part to the challenges of modeling UFP [17–22], there is little epidemiological literature on the health consequences of long-term UFP exposure. In a cross-sectional study of the health effects of annual average particle number concentration (PNC) among adults in the Community Assessment of Freeway Exposure and Health Study residing in several near-highway and urban background areas of the greater Boston area, we found positive associations with high sensitivity C-reactive protein (hsCRP), a biomarker of systemic inflammation [23]. The few previously published longitudinal analyses of the long-term health effects of UFP considered UFP modeled with larger spatial resolutions (between 200 m and 4 km). One study found that UFP mass was associated with increased ischemic heart disease mortality [24]. Other studies of long-term exposure to PNC have found that PNC was positively associated with sub-clinical markers of atherosclerosis and was inconsistently associated with biomarkers of inflammation and with respiratory outcomes [25–27].

Given the small number of studies on the health effects of long-term UFP exposure, we investigated the relationship between UFP and cardiovascular risk factors in the prospective Boston Puerto Rican Health Study (BPRHS) [28]. In this population, proximity to traffic was previously associated with changes in hsCRP levels over two years [29]. For the present study, our primary objective was to assess whether ambient residential UFP number concentrations at a fine spatial scale (≤20 m resolution) were associated with BP and hsCRP levels over six years. We also explored a novel exposure metric, the particle inhalation rate (PIR, particles inhaled/time), that may more closely approximate the biologically-relevant dose of UFP.

## Methods

### Study population

**Fig. 1:**
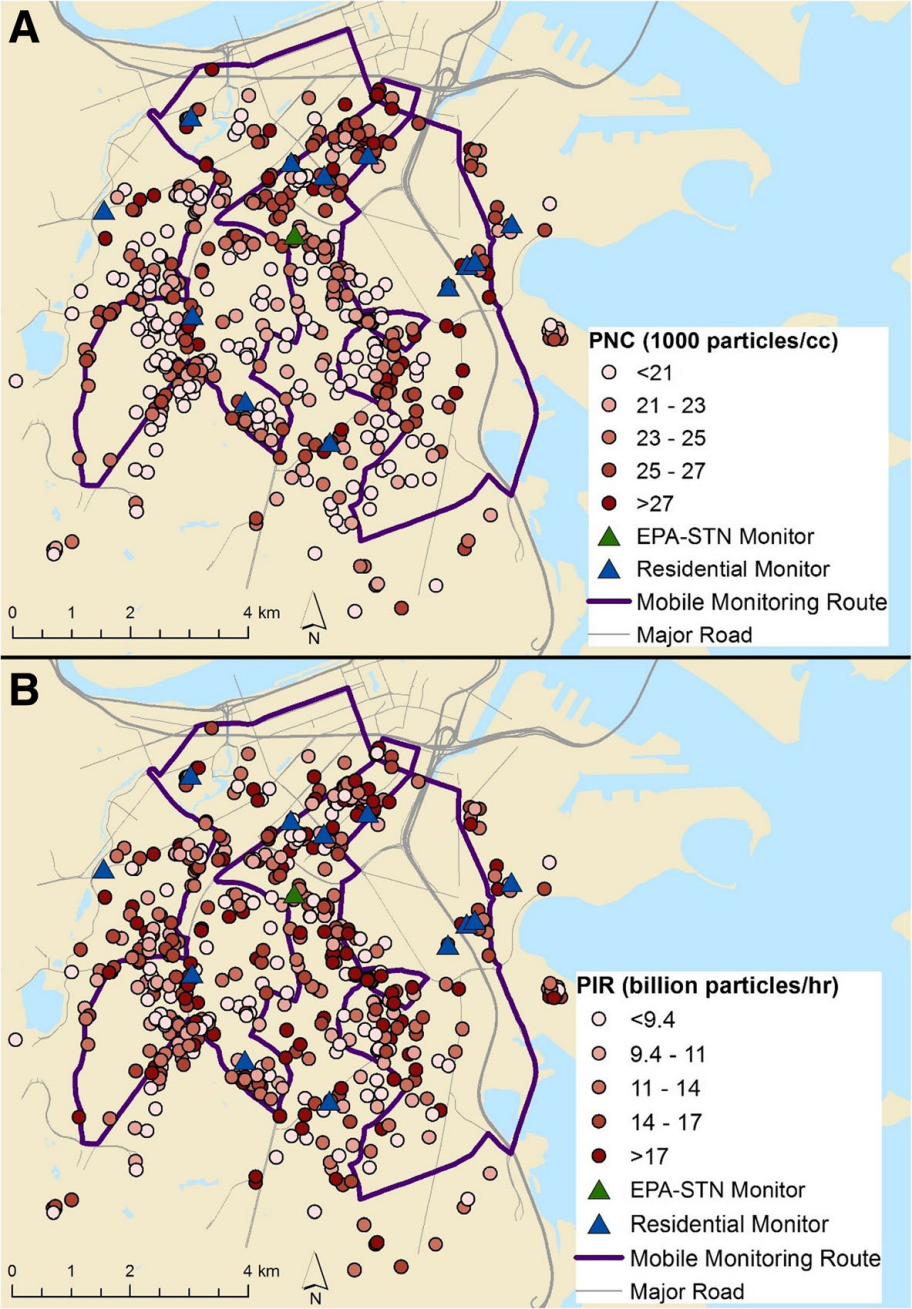
Spatial distribution of modeled annual average PNC and PIR. **a**) particle number concentration (PNC) and **b**) particle inhalation rate (PIR; by quintiles) at participant residences at baseline (*n* = 754). Data from mobile monitoring, a central monitor at a U.S. Environmental Protection Agency Speciation Trends Network (EPA-STN) site, and residential monitors were used to build and validate the PNC model

The BPRHS is a prospective cohort study of 1499 individuals designed to investigate the risk factors for chronic disease among Puerto Ricans living in eastern Massachusetts [28]. Briefly, participants were recruited through door-to-door enumeration and through community approaches from census tracts in the greater Boston area with at least 10 Hispanics aged 45–75 years. Individuals were eligible for inclusion in the BPRHS if they were 45–75 years old at baseline, were able to answer questions in English or Spanish, and self-identified as being Puerto Rican. Participants were excluded if they had plans to move within two years or if they had low cognitive function as measured by the Mini Mental State Examination (scores ≤10). We restricted our analyses to those participants who lived within a 1000 m buffer of our air pollution monitoring area at any study visit (*n* = 791, Fig. 1).

This study was approved by the Institutional Review Boards at Tufts Medical Center, Northeastern University, and the University of Massachusetts Lowell. All participants provided written informed consent.

### Health data

Participants were visited up to three times over approximately six years (visit one between 2004 and 2009, visit two between 2006 and 2011, and visit three between 2011 and 2015). The mean time between visit one and visit two was 2.2 years while the mean time between visit two and visit three was 4.1 years. Trained Spanish-English bilingual interviewers administered questionnaires in participants’ homes. Educational attainment was analyzed as a dichotomous variable (>8th grade/≤8th grade), based on the distribution of educational attainment in this population. Smoking status was assessed as current, former, or never (< 100 cigarettes smoked). Poverty status was determined by comparing participants’ total self-reported annual household income to the U.S. Census Bureau annual thresholds [30]. Medication use (prescription and over-the-counter) was assessed by visual inspection of medications. Physical activity was assessed using a modified Paffenbarger questionnaire of the Harvard Alumni Activity Survey which has been previously validated in an elderly Puerto Rican population [31, 32]. Validated scales were used to assess psychological acculturation and perceived stress [33, 34]. Participants were considered depressed if they reported taking medication for depression or if they scored ≥16 on the Center for Epidemiology Studies Depression Scale [35–37].

Height and weight were measured in duplicate. Body mass index (BMI) was calculated as kg/m^2^. A trained interviewer measured participants’ seated systolic blood pressure (SBP) and diastolic blood pressure (DBP) with an electronic sphygmomanometer (Dinamap™ Model 8260, Critikon, Tampa, FL), in duplicate, three times. The second and third sets of readings were averaged [28]. Participants were considered hypertensive if they had a SBP ≥140 mmHg, a DBP ≥90 mmHg, self-reported a diagnosis of hypertension, or if they were taking medication for hypertension. Pulse pressure (PP) was calculated as the difference between SBP and DBP.

A phlebotomist obtained blood samples after each study visit (> 81% had fasting samples at each visit; visits generally occurred in the morning). Cholesterol, triglycerides, serum glucose, and hsCRP concentrations were measured as described previously [28]. We excluded the top 1% of hsCRP values (≥47 mg/L) since extremely high hsCRP values are associated with acute infections [38]. Triglyceride and hsCRP concentrations were natural log transformed due to their skewed distributions. Participants were considered diabetic if their glucose concentration was ≥126 mg/dL, they were taking medications for diabetes, or they self-reported diabetes.

### Geolocation of participants’ residences

Participants’ residential addresses at each study visit were geocoded using ArcMap [39]. Addresses that could not be parcel-matched using the Boston parcel data were geocoded using Google Earth and publicly accessible site maps of housing developments. We geocoded 97% of participants’ residential locations at their second study visit (41% matched automatically) and 96% at their third study visit (39% matched automatically). A randomly selected subset of 12% of the geocoded locations for participants who moved was independently checked. All addresses were geocoded to the same parcel and the mean difference in position was less than 10 m.

### Exposure assessment

Exposure to UFP was estimated using a land-use regression model based on a previously published model [40]. Details on model development and validation are provided in the Additional file 1 (Part 1; see Table S1 for the full model). Briefly, the model was built using measured PNC from mobile and stationary platforms (Fig. 1) [41], meteorological data, and distances from specific roadways and bus routes (model-adjusted r^2^ = 0.37; see Additional file 1, Part 1) [42, 43]. The r^2^ is similar to other hourly PNC models specific to metropolitan Boston [44]. The model predicted hourly ambient PNC at each participant’s address with ≤20 m resolution. For all PNC estimates before 2012, the hourly model was back-extrapolated using meteorological data collected during the time period of interest. This was possible because PNC regression models are largely stable over time [45] and models of traffic-related air pollutants, including PNC, perform reasonably well when back-extrapolated [46, 47]. Each participant was then assigned an ambient annual average PNC corresponding to the 365 days immediately preceding each of their study visits.

### Calculation of particle inhalation rate

The particle inhalation rate (PIR, number of particles inhaled/h) was estimated for each participant as the product of the annual average PNC estimate (particles/L) and the hourly respiratory volume (tidal volume * breaths/h = L of air inhaled/h). We used published estimates for age- and sex-specific minute respiratory volume (L of air inhaled/min-kg) adjusted for weight and physical activity [48] together with data on how many hours per typical weekday and weekend day participants engaged in various levels of physical activity (lying down, sitting, light activity, moderate activity, and vigorous activity; for algorithm see Additional file 1, Part 2).

### Statistical analysis

For each outcome (SBP, DBP, PP, ln(hsCRP)), we developed two different multilevel linear models to consider the longitudinal associations of 1) PNC and 2) PIR with the levels of the outcomes across study visits. All models controlled for age, included a random intercept for each participant, and used data from every completed visit. All modeling was performed in Stata v14 [49]. To facilitate direct comparisons between the PNC and PIR models, we scaled results to the inter-quartile range (IQR; 4.6 thousand particles/mL and 6.2 billion particles inhaled/h, respectively).

We used a multi-stage process to select covariates. From an initial set of potential covariates identified by a literature review, we first included variables in the models if they were 1) associated with the outcome (*p* < 0.15) in bivariate analysis, and 2) either associated with the air pollution measure (*p* < 0.15) or changed the effect estimate for the air pollution measure by ≥10% in models that included only the air pollution measure and the potential covariate. Variables were retained if they were associated with the outcome in the multivariate model (p < 0.15) and if they did not introduce problems with collinearity based on variance inflation factors and correlation coefficients. We then assessed the effect of adding other variables that were considered potentially important based on the literature but had not met our initial inclusion criteria. If these variables were not associated with the outcome (*p* < 0.15) and did not materially change the effect estimates for PNC or PIR, they were excluded. Model residuals and model fit statistics were examined after the addition of each new covariate. Time varying predictors considered during the covariate selection process included age, BMI, high-density lipoprotein (HDL) cholesterol, low-density lipoprotein (LDL) cholesterol, ln(triglycerides), diabetes, hypertension medication, smoking, anxiety medication, perceived stress, psychological acculturation, marital status, and physical activity. Time invariant predictors considered included sex, educational attainment, and year of baseline visit. Year of baseline visit was included if it was a significant predictor to account for annual trends not captured within the PNC model. Physical activity and sex were not considered as confounders in the PIR models, as they were used in the calculation of the exposure. Variables assessed only at the third study visit (e.g., secondhand smoke exposure, family history of hypertension) were included only in sensitivity analyses and were assumed to be time-invariant. For each model, we checked collinearity and intra-class correlations. We also checked the normality and homoscedasticity of the residual errors.

Based on evidence from previous studies [1], we examined effect modification by sex, medication use, family history of hypertension (for BP), family history of CVD (for hsCRP), diabetes, smoking, employment status at baseline, physical activity, age, and BMI. To account for the high prevalence of baseline hypertension and cardiovascular disease, we also considered the effect of using the baseline measure of the outcome as a covariate in models that only used outcome data from the second and third study visits. Additionally, we conducted sensitivity analyses of the main models excluding participants who did not complete all three study visits, including participants with hsCRP values >99th percentile and excluding participants who, at baseline, self-reported at least one previous heart attack or stroke, had hypertension, had high baseline hsCRP (> 3 mg/L), or who died before their third study visit (*n* = 50).

## Results

Sixty-nine percent of participants were female and about half had attained more than an eighth grade education (Table 1). At each study visit, more than 70% of participants reported a household income below 120% of the federal poverty line [50] and only 22.1% of participants were employed at baseline. At baseline, 44.4% of participants reported never smoking, 23.7% were current smokers, and 31.9% were former smokers. Although the mean age at baseline was 57.1 years (standard deviation = 7.4; Table 1), 11.2% of participants had suffered at least one heart attack or stroke, 72.3% had hypertension, 46.0% had diabetes, 37.3% took antilipidemic (statin) medications, and 65.7% showed depressive symptomology. The participants included in the present analysis were similar to the larger BPRHS population [28].

**Table 1:** Participant characteristics by study visit

	Visit One		Visit Two		Visit Three	
	N	Mean (s) or %	N	Mean (s) or %	N	Mean (s) or %
SBP (mmHg)	731	134.6 (18.8)	600	136.8 (19.2)	423	135.0 (18.4)
DBP (mmHg)	730	80.9 (10.6)	600	80.4 (10.6)	423	75.4 (10.4)
PP (mmHg)	730	53.7 (14.6)	600	56.4 (15.8)	423	59.7 (16.3)
hsCRP (mg/L)	727	6.25 (9.02)	576	6.48 (11.04)	387	7.04 (9.49)
Age	754	57.1 (7.4)	605	59.2 (7.5)	431	63.1 (7.3)
BMI (kg/m^2^)	747	31.7 (6.3)	585	31.6 (6.5)	395	31.0 (6.7)
HDL (mg/dL)	737	44.2 (12.3)	594	46.5 (12.6)	395	47.3 (15.7)
LDL (mg/dL)	721	107.5 (35.5)	585	109.7 (35.5)	392	105.5 (35.1)
Triglycerides (mg/dL)	737	164 (114)	594	154 (93)	395	146 (109)
Physical activity score	751	31.8 (5.0)	603	31.3 (4.5)	429	31.7 (6.2)
Perceived stress	751	22.9 (9.6)	603	22.5 (9.0)	424	28.2 (7.2)
Distance from nearest interstate highway (m)^a^	754	1800 (1120)	605	1770 (1100)	409	1710 (1070)
Distance from nearest major road (m)^b^	754	240 (230)	605	240 (230)	409	250 (240)
Inhalation rate (L/h)	747	580 (220)	588	560 (200)	395	570 (230)
Female (%)	515	68.3	428	70.7	307	71.6
>8th grade education (%)	396	52.9	314	52.1	216	50.6
Household income < 120% poverty line (%)	504	71.3	427	75.2	291	77.6
Current smoker (%)	178	23.7	133	22.0	78	18.8
Former smoker (%)	240	31.9	199	33.0	158	38.1

^a^Interstate highways 90 or 93

^b^Major roads are defined as carrying ≥10,000 vehicles per day

Exposure distributions are summarized in Table 2 and spatial distributions are shown in Fig. 1. The distributions of both PNC and PIR were fairly stable across study visits (Table 2). PNC and PIR values were significantly correlated (*r* = 0.356, *p* < 0.001).

**Table 2:** Exposure distributions for PNC and PIR

	PNC (1000 particles/mL)				PIR (1 billion inhaled/h)			
	Total	Visit 1	Visit 2	Visit 3	Total	Visit 1	Visit 2	Visit 3
Observations	1790	754	605	431	1730	747	588	395
Mean	23	24	23	23	13	14	13	13
Standard deviation	3.4	3.5	3.1	3.5	5.5	5.6	5.0	6.0
Minimum	8.1	11	10	8.1	3.7	3.7	3.9	4.0
25th percentile	21	21	22	21	9.5	9.9	9.4	8.8
Median	24	24	24	24	12	13	12	12
75th percentile	26	26	26	26	16	16	15	15
Maximum	32	32	31	30	54	54	37	39
Range	24	21	20	22	51	51	33	35
IQR	4.6	5.0	4.2	5.0	6.2	6.3	6.0	6.6

**Fig. 2:**
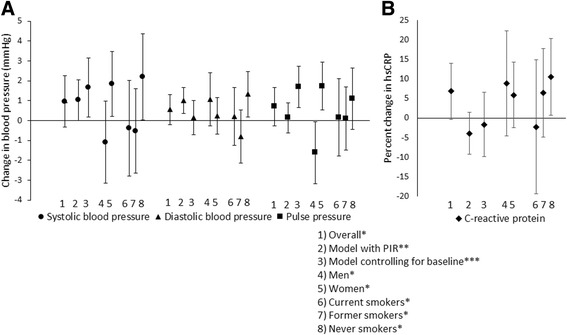
Associations between PNC and PIR with blood pressure and high sensitivity C-reactive protein. **a**) Change in blood pressure (mmHg) and **b**) percent change in C-reactive protein (mg/L) with an inter-quartile increase in PNC or PIR. All models control for age. Models additionally adjusted for: * Systolic blood pressure (SBP): education, sex, BMI, high-density lipoprotein (HDL) cholesterol, ln(triglycerides), hypertension medication, anxiety medication, marital status, and year of baseline visit; Diastolic blood pressure (DBP): sex, BMI, low-density lipoprotein (LDL) cholesterol, HDL cholesterol, ln(triglycerides), diabetes, marital status, and year of baseline visit; Pulse pressure (PP): education, LDL cholesterol, hypertension medication, diabetes, marital status, and smoking; High sensitivity C-reactive protein (hsCRP): education, sex, BMI, LDL cholesterol, HDL cholesterol, diabetes, anxiety medication, and smoking. ** SBP: education, BMI, LDL cholesterol, HDL cholesterol, ln(triglycerides), hypertension medication, anxiety medication, marital status, and year of baseline visit; DBP: BMI, LDL cholesterol, HDL cholesterol, ln(triglycerides), marital status, smoking, and year of baseline visit; PP: education, LDL cholesterol, hypertension medication, diabetes, marital status, and smoking; hsCRP: education, BMI, LDL cholesterol, HDL cholesterol, ln(triglycerides), diabetes, and anxiety medication. *** The same covariates are used as in the main models with PNC but only outcome data from the second and third study visits are included

Overall, long-term exposure to PNC was not associated with SBP (β = 0.96; 95% CI = − 0.33, 2.25 mmHg per 4600 particles/mL; Fig. 2). Sex modified the effect of PNC on SBP. Among females, PNC was positively associated with SBP (β = 1.84; 95% CI = 0.21, 3.48 mmHg) while for males, PNC was inversely but not significantly associated with SBP (β = − 1.09; 95% CI = − 3.16, 0.98 mmHg). Additionally, PNC was positively associated with SBP among never smokers (β = 2.20; 95% CI = 0.04, 4.37 mmHg), but not among current or former smokers (Additional file 1: Table S2). When controlling for the baseline level of SBP, PNC was associated with changes in SBP (β = 1.66; 95% CI = 0.17, 3.14 mmHg). When using the PIR in place of PNC, exposure was associated with SBP (β = 1.03; 95% CI = 0.00, 2.06 mmHg per 6.2 billion particles inhaled/h). These associations were stronger among generally healthier participants (Additional file 1: Table S3). The associations between PIR and BP were attenuated when people with hypertension at baseline and, separately, people who dropped out before their third study visit were excluded (β = 1.12, 95% CI = − 0.49, 2.73 mmHg; β = 0.36, 95% CI = − 0.93, 1.64 mmHg, respectively).

There was no association overall between PNC and DBP (β = 0.55; 95% CI = − 0.20, 1.30 mmHg per 4600 particles/mL), except among never smokers (β = 1.32; 95% CI = 0.19, 2.46 mmHg; Fig. 2). Additionally, excluding people taking respiratory medications made the association between PNC and DBP levels significant (β = 1.03, 95% CI = 0.16, 1.89 mmHg). Nevertheless, controlling for the baseline level of DBP did not substantially affect the effect estimates (β = 0.15; 95% CI = − 0.72, 1.02 mmHg). In contrast, the PIR was associated with DBP (β = 1.01; 95% CI = 0.36, 1.66 mmHg per 6.2 billion particles inhaled/h). The positive associations between PIR and DBP were strongest among males, participants employed at baseline, and older participants (Additional file 1: Table S3). The associations between PIR and DBP were attenuated when excluding people with hypertension at baseline and, separately, excluding people who dropped out before their third study visit (β = 1.01, 95% CI = − 0.05, 2.07 mmHg; β = 0.64, 95% CI = − 0.18, 1.46 mmHg, respectively).

Similarly to the trends for SBP, PNC was not associated with PP overall (β = 0.70; 95% CI = − 0.27, 1.67 mmHg per 4600 particles/mL) but there was effect modification by sex (Fig. 2). Among females, PNC was positively associated with PP (β = 1.73; 95% CI = 0.52, 2.93 mmHg) while for males, PNC was inversely associated with PP (β = − 1.62; 95% CI = − 3.18, − 0.05 mmHg). The positive associations between PNC and PP were generally stronger among never smokers, participants who were not employed at baseline, the least physically active participants, older participants, non-diabetics, and participants with a family history of hypertension (Additional file 1: Table S2). When controlling for the baseline level of PP, PNC was associated with changes in PP (β = 1.69; 95% CI = 0.66, 2.72 mmHg). When using the PIR in place of PNC, however, exposure was not associated with PP (β = 0.14; 95% CI = − 0.62, 0.90 mmHg per 6.2 billion particles inhaled/h).

In contrast to the blood pressure measures, exposure to PNC was associated with 6.8% higher hsCRP overall (95% CI = − 0.3, 14.0% per 4600 particles/mL; Fig. 2). The associations were strongest among never smokers, people without a family history of CVD, and people taking statins (Additional file 1: Table S2). Including individuals with a hsCRP value ≥47, excluding individuals with a baseline hsCRP value of ≥3 mg/L, excluding individuals with a non-fasting baseline blood draw, or excluding individuals who dropped out before their third study visit all attenuated the association between PNC and levels of hsCRP (β = 6.3%, 95% CI = − 1.3, 14.0%; β = 2.8%, 95% CI = − 5.8, 11.4%; β = 5.6%, 95% CI = − 2.1, 13.4%; β = 5.0%, 95% CI = − 3.7, 13.6%, respectively). Additionally, in models controlling for the baseline level of hsCRP, PNC was not associated with changes in hsCRP (β = − 1.7; 95% CI = − 9.9, 6.5%). The PIR was also not associated with hsCRP overall (β = − 4.0; 95% CI = − 9.4, 1.3% per 6.2 billion particles inhaled/h), although there was an inverse association between PIR and hsCRP among diabetics, participants without a family history of CVD, and participants who were not taking statins (Additional file 1: Table S3).

Sensitivity analyses excluding people who died or excluding those who had a previous heart attack or stroke did not materially change the results (data not shown). Excluding hypertension medication as a covariate from the models did not materially change any of the results (data not shown).

## Discussion

Our study is one of only a few longitudinal studies to consider the health effects of long-term exposure to UFP, and it is the first to do so with UFP measured at high spatial and temporal resolution. Our primary finding is that annual average PNC exposure was positively associated with hsCRP but was not associated with the blood pressure measures overall. Sex and smoking status modified the associations of PNC and the outcomes with generally stronger positive associations among women and never smokers. In sub-populations where PNC affected blood pressure, the effect estimates were modest in terms of clinical significance. An IQR increase in PNC exposure was associated with increases in blood pressure approximately equivalent to the increase of blood pressure seen with an additional year of age [51].

The association between PNC and hsCRP was somewhat stronger than the associations with blood pressure despite the fact that 56% of participants had hsCRP values > 3 mg/L at baseline (indicative of high cardiovascular risk) [52]. Each IQR increase in PNC was associated with a 7% higher mean hsCRP concentration (95% CI = − 0.3, 14.0%). This positive association is consistent with the only other longitudinal study examining the association between PNC and hsCRP [26]. Similarly, a cross-sectional study among a different population in the Boston metropolitan area found positive associations between annual average PNC exposure and hsCRP [23]. Furthermore, our finding that the associations of PNC with hsCRP and with blood pressure were stronger among never smokers compared to current or former smokers is consistent with the PM literature [53]. This might be explained by the constant low-grade inflammation that smokers experience [54].

Although toxicological evidence suggests that UFP may exert cardiovascular effects, likely through mechanisms mediated by oxidative stress, inflammation, and endothelial dysfunction [55–57], the epidemiologic evidence is less clear regarding associations between UFP and cardiovascular risk factors. The vast majority of the previous epidemiological literature has only considered acute health effects or short-term exposure to UFP and the results have been inconsistent [58–66]. Our reported results do not account for potential acute changes in the outcome measures due to changes in short-term exposures. While it is possible that there are modest acute effects, a recent longitudinal study suggested that short-term effects did not substantially change the effect estimates for long-term exposures to PNC [26]. Moreover, within our data, including short-term effects could affect the performance of the models due to the high correlation between the short- and long-term exposures. Furthermore, if short-term effects aggregate to the long-term effects, including the short-term effects may not be conceptually valid as the short-term effects would be on the causal pathway [67].

One of the innovations of our study was the use of an exploratory exposure metric, the PIR. We developed an algorithm to calculate PIR in a longitudinal study based on an established framework [68] and using validated age-, sex-, weight- and physical activity level-specific estimates for hourly respiratory volume [48]. In a previous study, PIR was calculated by multiplying PNC by the amount of air inhaled per minute [68]. That study found different exposure trends compared to studies that did not account for inhalation [69, 70]. Neglecting inhalation rate may thus introduce exposure misclassification.

Nevertheless, there are fundamental differences in what PNC and PIR attempt to quantify. For 3% of observations, participants had a high PIR (>75th percentile) but low PNC exposure (<25th percentile) and a high average inhalation rate (>75th percentile). For these people, the particle deposition fraction and clearance rate may be the most relevant factors in relation to health effects [7, 71]. This is particularly important for certain sub-populations who have higher inhalation rates, such as males and those with greater physical activity. In our study, we found some evidence that associations between the PIR and CVD risk factors were stronger among males than females. Additionally, as may be expected if there were a true association between UFP and DBP levels, the association between the PIR and DBP in males was stronger than the association between PNC and DBP in males.

We might expect people who inhale more particles per hour to have increased susceptibility to high ambient concentrations of air pollutants, especially if individuals are exercising in highly polluted areas [72]. It is also possible that these people may be less vulnerable to health effects from air pollution if the higher PIR is due to greater levels of physical activity since exercise is associated with better cardiovascular health and the beneficial effect of exercise tends to outweigh the negative effect of exposure to air pollution [72, 73]. This was partially supported as the effect estimates for PNC and PIR were slightly stronger among the most sedentary participants in certain models. This trend was not entirely consistent, however, and in some cases, healthier individuals in our study appeared to be more susceptible. It is also possible that it was easier for us to observe associations among people who are not taking medications, such as statins, that could counteract the negative effects of PM exposure [74, 75].

Further work is needed to refine our PIR algorithm. In particular, it would be useful to refine the metric based on indoor measurements of UFP concentrations or based on an accounting of how much time participants spend in different indoor and outdoor micro-environments. Additionally, we may be over-accounting for weight or physical activity since including BMI as a covariate in the models for hsCRP changed the direction of the associations. This trend was not apparent in the BP models. Finally, it might be interesting to see if using age-, sex-, weight- and physical activity level estimates for hourly respiratory volume specific to this population (rather than from the EPA Exposure Factors Handbook) changes the results. While this may be expected if the health status of our participants differs substantially from that of the reference population, limiting our analyses to only those participants who were not taking respiratory medications and who did not have extremely high physical activity levels did not substantially change the effect estimates for the PIR. We believe that further developing the PIR metric is worthwhile since it addresses a critical step on the exposure pathway and may reduce exposure misclassification.

Our study had several limitations. One was the temporal mismatch between our exposure monitoring and participant visits. Participant visits occurred between 2004 and 2015 while we monitored UFP concentrations from December 2011 through November 2013. During this time, UFP emissions could have changed. Nevertheless, much of the temporal variability in PNC exposure is explained by meteorological conditions and we have historical data for these parameters [76, 77]. Additionally, modeled estimates compared reasonably well to PNC measurements at a stationary site for the years in which the model was back-extrapolated (see Additional file 1, Part 1). Annual trends were also accounted for in the health association models if the term for the year participants started the study was a significant predictor.

Other limitations of our exposure assessment include our assumptions that spatial variability was constant with time, that the model is valid up to 1000 m from the monitoring area, and that participants’ residential annual average PNC was representative of their overall personal exposure. Although we do not have time-activity data available in the BPRHS, we have shown in previous work that accounting for time-activity could reduce exposure misclassification [78]. We also assumed that observed associations were due to long-term UFP exposure without accounting for potential interactions with, or independent effects of, other traffic-related pollutants or traffic-related noise. It was not possible to account for other traffic-related pollutants because we do not currently have exposure models for any other pollutants. Additionally, while our assumption that a one year averaging period represents a biologically relevant time-frame is in accordance with much of the related literature on long-term exposure to traffic-related air pollutants [2, 4], it is possible that we did not capture the critical averaging window for UFP. It is also possible that short-term effects would change the effect estimates for the long-term exposures in this study even though they did not in a previous longitudinal study [26].

Limitations also included the substantial attrition and resulting potential for selection bias. While only 49% of the 791 participants contributed data at all three time points, the baseline characteristics of participants who stayed in the study were similar to those who dropped out and most of the results were not substantially affected by excluding individuals who completed fewer than three study visits. Furthermore, our results may not be generalizable to healthier populations. All of our study participants were Puerto Rican, most had low socioeconomic status, and most had at least one chronic health condition at baseline. Other potential limitations of our analysis include the large number of comparisons and resulting possibility that some significant findings are due to chance, relatively low exposure contrast across the study population possibly limiting our ability to find true associations, the potential for misclassification of covariates such as diabetes, and the potential for residual confounding.

## Conclusions

We found that both PNC and PIR were associated with biomarkers of CVD risk over six years, although the trends were not entirely consistent. The PIR is a novel exposure metric that accounts for differential inhalation rate. As our study is among the first to address these questions, future work is needed to validate these findings.

## Additional file

Additional file 1: Part 1. Exposure assessment and **Table S1.** PNC model used for exposure assignment; Part 2. Inhalation rate adjustment; Part 3. **Table S2.** Longitudinal associations with an IQR increase in PNC (4600 particles/mL) and **Table S3.** Longitudinal associations with an IQR increase in PIR (6.2 billion particles inhaled/h). (DOCX 62 kb)

## Abbreviations

BMI: Body mass index
BP: Blood pressure
BPRHS: Boston Puerto Rican Health Study
CI: Confidence interval
CVD: Cardiovascular disease
DBP: Diastolic blood pressure
HDL: High-density lipoprotein
hsCRP: High sensitivity C-reactive protein
IQR: Inter-quartile range
LDL: Low-density lipoprotein
PIR: Particle inhalation rate
PM: Particulate matter
PNC: Particle number concentration
PP: Pulse pressure
SBP: Systolic blood pressure
UFP: Ultrafine particulate matter
