# Supplementary material for: Longitudinal associations of long-term exposure to ultrafine particles with blood pressure and systemic inflammation in Puerto Rican adults
Source: Environ Health. 2018 Apr 5;17:33. doi: 10.1186/s12940-018-0379-9 (PMC5887259; doi:10.1186/s12940-018-0379-9)
Supplement: Supplementary file 1 — Part 1. Exposure assessment and Table S1. PNC model used for exposure assignment; Part 2. Inhalation rate adjustment; Part 3. Table S2. Longitudinal associations with an IQR increase in PNC (4600 particles/mL) and Table S3. Longitudinal associations with an IQR increase in PIR (6.2 billion particles inhaled/h). (DOCX 62 kb) [file 12940_2018_379_MOESM1_ESM.docx]

**Supplement Table of Contents**

Section 1. Exposure assessment 2

Table S1. PNC exposure model 4

Section 2. Inhalation rate adjustment 6

Section 3. Supplemental tables 7

Table S2. Longitudinal associations with PNC 7

Table S3. Longitudinal associations with PIR 8

Section 4. References 11

**Supplement Section 1. Exposure Assessment**

PNC Monitoring

Ambient particle number concentration (PNC) was monitored continuously between December 2011 and November 2013 at a U.S. Environmental Protection Agency Speciation Trends Network site (EPA-STN, Figure 1 of main text). Measurements were made using a water-based condensation particle counter (CPC, model 3783, TSI, Shoreview, MN, D_50_ = 7 nm) every minute before May 2013 and every 30 seconds thereafter. Measurements were averaged to the hour. During this period, mobile monitoring of PNC was also conducted with the Tufts Air Pollution Monitoring Laboratory (TAPL). The TAPL is a recreational vehicle retrofitted with rapid-response gas and particle phase instruments [1]. Monitoring was conducted in 3-6 hour sessions over 49 days between December 2011 and November 2013 along a fixed ~40 km route (~40 km² area; Figure 1). In each season, monitoring sessions captured traffic and meteorological conditions between 05:00 and 21:00 on both weekdays and weekends. The TAPL was driven on non-highway streets at 5-10 m/s so that local-scale changes in PNC could be measured. A butanol-based CPC (model 3775, TSI, D_50_ = 4 nm) was used to measure PNC each second. A Garmin eTrex GPS (manufacturer-specified accuracy: 3–5 m) was used as a master clock to match instrument times so that spatial coordinates could be assigned. Quality control protocols have been described previously [2]. Both CPCs were collocated in the laboratory for a side-by-side analysis and they showed good agreement (r^2^ = 0.94).

PNC Modeling

Modeling was based on a previously developed land use regression model for a different part of the Boston metropolitan area [2]. The covariates used were largely the same but a new model was developed since the model in Patton et al. (2014) was neighborhood specific and it has been shown that transferability of these models between neighborhoods is limited [3]. Additionally, our model was able to better account for temporal patterns of PNC within the study area since we included data from the EPA-STN site which was not used previously. Models for PNC were developed in R using PNC measurements from both the stationary and mobile platforms. An hourly temporal model (Model A) was developed first from measurements at the EPA-STN site and meteorological data obtained from Logan International Airport in Boston (Table S1) [4]. Covariates were selected based on a stepwise linear regression method as described in Patton et al. (2014). Model A predictions were then subtracted from the TAPL measured spatio-temporal PNC data. The remaining spatial residuals (and error) were used to build a spatial PNC model (Model B; Table S1). Variables tested in Model B included land-use characteristics and distances to major roadways and point sources. Distance variables assigned to participants were calculated separately for each study visit to account for changes in participants’ residential locations. All distance variables were calculated in ArcGIS v10.1. The sum of Models A and B provided the overall PNC exposure estimate for each participant.

Table S1. PNC model used for exposure assignment.*

|  | **Covariate (Unit)** |  | **Beta** | **Standard Error** |
| --- | --- | --- | --- | --- |
| **Model A (Temporal)** | Intercept |  | 14.93 | 0.664 |
|  | Temperature (K) |  | -0.0255 | 0.0005 |
|  | Relative Humidity (%) |  | -0.0063 | 0.0003 |
|  | Day/Night (dichotomous factor) |  | 0.317 | 0.011 |
|  | Atmospheric Pressure (mmHg) |  | 0.053 | 0.011 |
|  | Atmospheric Pressure – lag 1 hr (mmHg) |  | -0.049 | 0.011 |
|  | Wind Speed (m/s) |  | -0.051 | 0.002 |
|  | Morning Rush (6-9am; dichotomous factor) |  | 0.201 | 0.015 |
|  | Evening Rush (4-7pm; dichotomous factor) |  | 0.077 | 0.014 |
|  | *Wind Direction (categorical, rel. ENE)* |  |  |  |
|  | ESE |  | -0.297 | 0.020 |
|  | SSE |  | -0.283 | 0.023 |
|  | SSW |  | -0.219 | 0.018 |
|  | WSW |  | -0.118 | 0.019 |
|  | WNW |  | -0.152 | 0.019 |
|  | NNW |  | -0.244 | 0.019 |
|  | NNE |  | -0.156 | 0.021 |
| **Model B (Spatial)** | Intercept |  | 0.418 | 0.004 |
|  | Within 10 m Major Road (dichotomous factor) |  | 0.074 | 0.004 |
|  | Distance to nearest bus route (m) |  | -1.76x10^-3^ | 2.13x10^-5^ |
|  | Distance to nearest interstate (m) |  | -2.22x10^-5^ | 1.24x10^-6^ |
|  | Distance to nearest road with ≥10,000 vehicles/day annual average (m) |  | -1.51x10^-4^ | 7.05x10^-6^ |

*The sum of the results from Models A and B represent the overall hourly exposure estimate for each participant in units of ln(particles/cm^3^). All terms were significant at p < 0.001 level.

The model was stable during 10-fold, 10-repeat cross validation. Additionally, the ability of the model to predict across the study area was validated by comparing modeled PNC to ambient PNC measured hourly at 10 participant homes throughout the study area (while the model was used to predict PNC at all participant homes in the study area, only 10 homes had PNC data available for comparison). Monitoring was conducted at these 10 sites for up to six weeks each between May 2012 and November 2013. The mean Pearson correlation coefficient of hourly modeled and measured ln(PNC) at participant homes was 0.44 (values between 0.36 and 0.51). The ability of the model to predict over time was tested by comparing modeled hourly PNC estimates to hourly PNC measured between 2003 and 2013 at the Harvard Clean Air Research Center (Figure 1) [5]. The comparison suggested that the model could predict reasonably well (r² = 0.36) outside of the timeframe from which the model was developed. Our hourly model performed similarly to a daily model, which compared modeled PNC and measured PNC at sites across the Netherlands 10 years prior to model development (r^2^ = 0.36) [6].

**Supplement Section 2. Inhalation Rate Adjustment Algorithm**

We used age-, sex-, weight-, and physical activity level-specific estimates for hourly respiratory volume [7] and modeled ambient residential PNC exposure to estimate each participants’ particle inhalation rate (PIR). As an example, for a male participant in his 50s, the algorithm would is as follows:

Step 1. Average number of liters of air breathed in one hour =

[(5 week days * body weight in kg * 60 minutes per hour)
*(0.07*number of hours sleeping or lying down during a weekday
+ 0.07*number of sedentary hours during a weekday
+ 0.17*number of hours of light physical activity during a weekday
+ 0.38*number of hours of moderate physical activity during a weekday
+ 0.68*number of hours of vigorous physical activity during a weekday)
+ (2 weekend days * body weight in kg * 60 minutes per hour)
*(0.07*number of hours sleeping or lying down during a weekend day
+ 0.07*number of sedentary hours during a weekend day
+ 0.17*number of hours of light physical activity during a weekend day
+ 0.38*number of hours of moderate physical activity during a weekend day

+ 0.68*number of hours of vigorous physical activity during a weekend day)]
/(7 days of the week * 24 hours)

Step 2. Average number of particles inhaled hourly (PIR) = PNC (particles/mL) * 1000 (mL/L) * average number of liters of air inhaled hourly.

**Supplement Section 3. Supplemental Tables**

Table S2. Longitudinal associations with an IQR increase in PNC (4600 particles/mL)

|  | SBP (mmHg)  β (95% CI; n) | DBP (mmHg)  β (95% CI; n) | PP (mmHg)  β (95% CI; n) | hsCRP (% change)  β (95% CI; n) |
| --- | --- | --- | --- | --- |
| Adjusted only for age | 1.32 (0.05, 2.58; 787) | 0.51 (-0.23, 1.25; 787) | 1.01 (0.03, 1.99; 787) | 9.3 (1.8, 16.7; 766) |
| Fully adjusted† |  | | | |
| Total sample | 0.96 (-0.33, 2.25; 762) | 0.55 (-0.20, 1.30; 757) | 0.70 (-0.27, 1.67; 760) | 6.8 (-0.3, 14.0; 749) |
| Sex |  |  |  |  |
| Male | -1.09 (-3.16, 0.98; 239) | 1.07 (-0.26, 2.40; 238) | -1.62 (-3.18, -0.05; 238) | 8.8 (-4.6, 22.2; 234) |
| Female | 1.84 (0.21, 3.48; 523) | 0.24 (-0.68, 1.15; 519) | 1.73 (0.52, 2.93; 522) | 5.8 (-2.6, 14.1; 515) |
| Smoker status†† |  |  |  |  |
| Current | -0.38 (-2.79, 2.03; 200) | 0.20 (-1.25, 1.65; 198) | 0.16 (-1.78, 2.10; 198) | -2.3 (-19.5, 14.8; 196) |
| Former | -0.53 (-2.66, 1.60; 279) | -0.81 (-2.13, 0.52; 276) | 0.10 (-1.48, 1.68; 277) | 6.4 (-4.9, 17.7; 270) |
| Never | 2.20 (0.04, 4.37; 335) | 1.32 (0.19, 2.46; 334) | 1.09 (-0.45, 2.63; 336) | 10.5 (0.7, 20.3; 333) |
| Baseline employment status |  |  |  |  |
| Yes | 0.98 (-1.77, 3.73; 152) | 1.31 (-0.30, 2.91; 151) | -0.62 (-2.55, 1.32; 151) | 4.2 (-9.3, 17.6; 149) |
| No | 0.36 (-1.15, 1.87; 520) | 0.33 (-0.54, 1.20; 516) | 0.46 (-0.70, 1.63; 520) | 4.9 (-4.1, 13.8; 512) |
| Family history of HTN (or CVD for hsCRP) |  |  |  |  |
| Yes | 0.89 (-1.01, 2.79; 323) | 0.19 (-0.85, 1.23; 321) | 1.18 (-0.22, 2.59; 321) | 0.0 (-10.9, 10.9; 278) |
| No | 0.65 (-2.84, 4.13; 85) | 0.49 (-1.33, 2.31; 85) | -0.13 (-2.87, 2.62; 85) | 12.2 (-2.4, 26.9; 124) |
| HTN medication use (or CVD for hsCRP) |  |  |  |  |
| Yes | 0.65 (-1.02, 2.31; 476) | 0.83 (-0.14, 1.79; 474) | 0.29 (-1.01, 1.59; 477) | 7.5 (-1.4, 16.3; 522) |
| No | 0.55 (-1.23, 2.33; 395) | -0.18 (-1.27, 0.92; 389) | 0.94 (-0.40, 2.28; 392) | 4.9 (-6.7, 16.5; 343) |
| Statin medication use |  |  |  |  |
| Yes | 0.68 (-1.25, 2.61; 408) | 0.82 (-0.30, 1.94; 405) | 0.30 (-1.17, 1.77; 411) | 10.0 (-0.6, 20.6; 392) |
| No | 0.87 (-0.80, 2.55; 518) | 0.41 (-0.62, 1.43; 511) | 0.80 (-0.40, 2.00; 515) | 0.1 (-8.9, 9.1; 511) |
| Diabetic |  |  |  |  |
| Yes | 0.10 (-1.64, 1.84; 390) | 0.69 (-0.40, 1.77; 388) | -0.17 (-1.46, 1.12; 388) | 6.8 (-4.4, 17.9; 381) |
| No | 1.20 (-0.67, 3.07; 437) | 0.20 (-0.81, 1.20; 434) | 1.07 (-0.32, 2.45; 435) | 7.3 (-2.1, 16.7; 430) |
| Baseline age |  |  |  |  |
| Younger than 65 years | 0.88 (-0.57, 2.32; 630) | 0.56 (-0.26, 1.38; 626) | 0.42 (-0.64, 1.48; 627) | 6.2 (-1.4, 13.9; 619) |
| At least 65 years | 1.19 (-1.76, 4.15; 132) | 0.15 (-1.86, 2.15; 131) | 2.48 (-0.09, 5.05; 133) | 12.1 (-7.7, 31.9; 130) |
| Physical activity |  |  |  |  |
| Quartile 1 | 1.97 (-0.63, 4.58; 290) | 0.25 (-1.52, 2.02; 284) | 2.48 (0.55, 4.41; 298) | 5.2 (-11.3, 21.6; 282) |
| Quartile 2 | -1.05 (-3.38, 1.28; 336) | -0.38 (-1.87, 1.10; 332) | -1.17 (-2.81, 0.48; 328) | 5.8 (-9.0, 20.6; 323) |
| Quartile 3 | 0.27 (-2.02, 2.56; 342) | 0.99 (-0.19, 2.17; 333) | -0.45 (-2.24, 1.34; 335) | 8.2 (-5.7, 22.2; 334) |
| Quartile 4 | 0.37 (-2.00, 2.74; 322) | 0.62 (-0.73, 1.96; 319) | -0.19 (-1.87, 1.48; 318) | 6.3 (-7.2, 19.8; 314) |

†SBP models adjusted for education, sex, BMI, high-density lipoprotein (HDL) cholesterol, ln(triglycerides), hypertension medication, anxiety medication, marital status, and year of baseline visit;

DBP models adjusted for sex, BMI, low-density lipoprotein (LDL) cholesterol, HDL cholesterol, ln(triglycerides), diabetes, marital status, and year of baseline visit;

PP models adjusted for education, LDL cholesterol, hypertension medication, diabetes, marital status, and smoking;

hsCRP models adjusted for education, sex, BMI, LDL cholesterol, HDL cholesterol, diabetes, anxiety medication, and smoking

††Total sample size in stratified analyses can exceed total overall if participants changed between visits (e.g., current smoker one visit, former smoker the next).

Table S3. Longitudinal associations with an IQR increase in PIR (6.2 billion particles inhaled/hr)

|  | SBP (mmHg)  β (95% CI; n) | DBP (mmHg)  β (95% CI; n) | PP (mmHg)  β (95% CI; n) | hsCRP (% change)  β (95% CI; n) |
| --- | --- | --- | --- | --- |
| Adjusted only for age | 1.23 (0.19, 2.26; 777) | 1.12 (0.49, 1.75; 777) | 0.14 (-0.60, 0.89; 777) | 7.1 (1.5, 12.7; 759) |
| Fully adjusted† |  | | | |
| Total sample | 1.03 (0.00, 2.06; 756) | 1.01 (0.36, 1.66; 759) | 0.14 (-0.62, 0.90; 756) | -4.0 (-9.4, 1.3; 749) |
| Sex |  |  |  |  |
| Male | 1.11 (-0.49, 2.71; 236) | 1.97 (1.05, 2.90; 239) | -0.68 (-1.90, 0.54; 237) | -3.0 (-12.4, 6.3; 234) |
| Female | -0.09 (-1.51, 1.33; 520) | -0.54 (-1.35, 0.28; 520) | 0.69 (-0.31, 1.69; 519) | 1.2 (-5.3, 7.7; 515) |
| Smoker status |  |  |  |  |
| Current | 1.14 (-1.08, 3.36; 197) | 1.29 (0.03, 2.55; 199) | -0.28 (-2.03, 1.47; 197) | -11.8 (-27.0, 3.5; 196) |
| Former | 0.90 (-0.97, 2.78; 277) | 1.15 (-0.06, 2.36; 280) | -0.11 (-1.36, 1.13; 275) | -7.0 (-15.8, 1.9; 270) |
| Never | 1.06 (-0.49, 2.62; 334) | 0.87 (-0.08, 1.83; 334) | 0.42 (-0.71, 1.56; 334) | 0.0 (-7.1, 7.1; 333) |
| Baseline employment status |  |  |  |  |
| Yes | 1.57 (0.00, 3.13; 151) | 1.39 (0.24, 2.53; 151) | 0.04 (-0.99, 1.07; 151) | 0.2 (-7.6, 7.9; 149) |
| No | 0.20 (-1.27, 1.66; 515) | 0.72 (-0.16, 1.59; 517) | -0.48 (-1.59, 0.63; 516) | -5.5 (-13.5, 2.4; 512) |
| Family history of HTN (or CVD for hsCRP) |  |  |  |  |
| Yes | 0.62 (-0.69, 1.93; 320) | 0.61 (-0.27, 1.50; 321) | 0.27 (-0.66, 1.21; 320) | -4.1 (-13.0, 4.8; 278) |
| No | -0.09 (-2.95, 2.78; 85) | 0.65 (-0.70, 2.00; 86) | -0.53 (-3.00, 1.95; 85) | -7.9 (-16.4, 0.6; 124) |
| HTN medication use (or CVD for hsCRP) |  |  |  |  |
| Yes | 0.10 (-1.36, 1.57; 473) | 1.09 (0.17, 2.00; 472) | -0.58 (-1.71, 0.54; 472) | -4.4 (-11.0, 2.2; 524) |
| No | 1.38 (0.02, 2.74; 391) | 0.99 (0.08, 1.91; 391) | 0.64 (-0.28, 1.56; 389) | -6.0 (-14.5, 2.5; 343) |
| Statin medication use |  |  |  |  |
| Yes | -0.31 (-2.20, 1.58; 406) | 0.99 (-0.17, 2.16; 405) | -0.71 (-2.15, 0.73; 403) | 0.0 (-8.7, 8.7; 394) |
| No | 1.79 (0.52, 3.05; 510) | 1.33 (0.53, 2.13; 512) | 0.52 (-0.32, 1.36; 511) | -6.6 (-13.2, 0.0; 511) |
| Diabetic |  |  |  |  |
| Yes | 0.79 (-0.74, 2.32; 387) | 1.13 (0.22, 2.04; 386) | 0.14 (-1.05, 1.34; 385) | -7.9 (-15.5, -0.3; 383) |
| No | 0.80 (-0.62, 2.22; 432) | 0.72 (-0.19, 1.64; 434) | 0.05 (-0.86, 0.97; 433) | -1.2 (-8.5, 6.0; 430) |
| Baseline age |  |  |  |  |
| Younger than 65 years | 1.03 (-0.01, 2.06; 625) | 0.98 (0.31, 1.66; 628) | 0.10 (-0.67, 0.86; 625) | -5.0 (-10.6, 0.6; 619) |
| At least 65 years | 1.11 (-3.64, 5.86; 131) | 2.06 (-0.31, 4.44; 131) | 0.60 (-2.61, 3.82; 131) | 5.8 (-13.9, 25.5; 130) |
| Physical activity |  |  |  |  |
| Quartile 1 | 5.29 (-0.16, 10.75; 287) | 4.14 (0.04, 8.24; 286) | 1.13 (-2.20, 4.46; 285) | -18.6 (-53.8, 16.5; 283) |
| Quartile 2 | 1.08 (-3.63, 5.79; 328) | 2.11 (-0.91, 5.14; 332) | -1.76 (-4.47, 0.95; 327) | -1.4 (-30.0, 27.2; 325) |
| Quartile 3 | 1.07 (-2.84, 4.97; 337) | 1.85 (-0.10, 3.80; 338) | 0.24 (-2.25, 2.73; 333) | -4.6 (-28.0, 18.8; 334) |
| Quartile 4 | 1.29 (-0.52, 3.09; 318) | 1.25 (0.09, 2.42; 320) | 0.09 (-1.25, 1.43; 316) | 3.6 (-6.6, 13.7; 315) |

†SBP models adjusted for education, BMI, LDL cholesterol, HDL cholesterol, ln(triglycerides), hypertension medication, anxiety medication, marital status, and year of baseline visit;

DBP models adjusted for BMI, LDL cholesterol, HDL cholesterol, ln(triglycerides), marital status, smoking, and year of baseline visit;

PP models adjusted for education, LDL cholesterol, hypertension medication, diabetes, marital status, and smoking;

hsCRP models adjusted for education, BMI, LDL cholesterol, HDL cholesterol, ln(triglycerides), diabetes, and anxiety medication

**Supplement Section 4. References**

1. Padró-Martínez LT, Patton AP, Trull JB, Zamore W, Brugge D, Durant JL. Mobile monitoring of particle number concentration and other traffic-related air pollutants in a near-highway neighborhood over the course of a year. Atmos Environ. 2012;61:253–64.

2. Patton AP, Collins C, Naumova EN, Zamore W, Brugge D, Durant JL. An Hourly Regression Model for Ultrafine Particles in a Near-Highway Urban Area. Environ Sci Technol. 2014;48:3272–80.

3. Patton AP, Zamore W, Naumova EN, Levy JI, Brugge D, Durant JL. Transferability and generalizability of regression models of ultrafine particles in urban neighborhoods in the Boston area. Environ Sci Technol. 2015;49:6051–60.

4. National Climatic Data Center. Land-Based Station Data [Internet]. 2015 [cited 2015 Mar 16]. Available from: http://www.ncdc.noaa.gov/data-access/land-based-station-data

5. Harvard Clean Air Research Center. Harvard Clean Air Research Program [Internet]. 2015 [cited 2015 Dec 7]. Available from: http://www.hsph.harvard.edu//clarc/index.html

6. Montagne DR, Hoek G, Klompmaker JO, Wang M, Meliefste K, Brunekreef B. Land Use Regression Models for Ultrafine Particles and Black Carbon Based on Short-Term Monitoring Predict Past Spatial Variation. Environ Sci Technol. 2015;49:8712–20.

7. US EPA. Metabolically Derived Human Ventilation Rates: A Revised Approach Based Upon Oxygen Consumption Rates (Final Report) 2009 [Internet]. 2009 [cited 2015 Jul 27]. Available from: http://cfpub.epa.gov/ncea/cfm/recordisplay.cfm?deid=202543
